# High surface recombination velocity limits Quasi-Fermi level splitting in kesterite absorbers

**DOI:** 10.1038/s41598-018-19798-w

**Published:** 2018-01-30

**Authors:** Alex Redinger, Thomas Unold

**Affiliations:** 1 Department Structure and Dynamics of Energy Materials, Helmholtz-Zentrum Berlin für Materialien und Energie GmbH, Glienicker Str. 100, 14109 Berlin, Germany; 2 Scanning Probe Microscopy Laboratory, Physics and Materials Science Research Unit, University of Luxembourg, 162a, avenue de la Faïencerie, L-1511 Luxembourg, Luxembourg

## Abstract

Kelvin Probe Force Microscopy, Photoluminescence imaging and numerical simulations are used to study the surfaces of Cu_2_ZnSnSe_4_ absorber layers. In particular, the effect of NH_4_OH and annealing under ambient conditions is investigated. We observe drastic changes in the measured quasi Fermi-level splitting (QFLs) after chemical cleaning of the absorber surface with NH_4_OH, which is traced back to a removal of the surface inversion. Air annealing recovers surface inversion, which reduces the recombination current at the surface. Annealing above 200 °C leads to a permanent change in the work function which cannot be modified by NH_4_OH etching anymore. This modification makes the QFLs insensitive to surface cleaning and explains why air annealing in Cu_2_ZnSnSe_4_ is important. From numerical simulations we find that a large surface recombination velocity needs to be present in order to describe the experimental observations.

## Introduction

The benefits of thin film solar cells are numerous. Material consumption, compared to crystalline silicon is strongly reduced and fabrication methods require less energy. The absorber layers are thin enough to be grown on flexible substrates and high power conversion efficiencies (>20%) have been reported for some material systems, such as Cu(In,Ga)Se_2_, CdTe and hybrid organic/inorganic perovskites^1^. Kesterite based solar cells are promising alternatives to the aforementioned materials systems. However, there is still a substantial gap in record power conversion efficiency between kesterites (12.6%^2^) and the high efficiency material systems. Consequently, there is a need to study kesterites in great detail and to identify the bottlenecks of this technology. One of the most challenging properties of thin film solar cells is their polycrystalline nature, which introduces a high level of difficulty and techniques with a high spatial resolution are necessary in order to measure grain dependent surface properties or grain boundaries (GBs). Kelvin Probe Force Microscopy (KPFM) is a powerful characterization technique to study the electrostatic landscape of semiconductors with nanometer resolution. In KPFM, a conductive tip is scanned over the surface of the absorber layer. Since the metallic tip and the semiconductor exhibit different work functions, a contact potential difference (CPD) forms. At each tip position a voltage is applied between the tip and sample which nullifies the CPD. As a result a CPD map of the surface is obtained. In the easiest case, changes in the CPD are equal to a changes in the work function of the material on the nanometer scale. This property is extremely important for solar cells, since it determines the band alignment at the heterojunction and at the GBs^3^. However, it has to be stressed that CPD variations also arise from changes in the surface dipole and due to differences in absorber doping, which results in a shift of the Fermi-level. In addition, secondary phases and adsorbates present on the surface may also change the CPD value, as different materials may have different surface properties.

KPFM has been used in a number of publications for the case of CIGSe^4,5^ and CdTe^6^. More recently, KPFM has also been used on kesterite absorbers and so far there is no consensus on the influence of the GBs in the solar cells. In the following we give a short summary of what has been published so far. One of the first studies have been published by Li *et al*.^7^ who measured CIGSe and CZTSSe absorbers with KPFM. The samples were measured in air after soaking the absorber with deionized water (DI) water for 1 h. The authors measured mostly downward band bending at the GBs on CIGSe and CZTSSe (smaller work function at the GB compared to the grain interior). Xin and coworkers^8^ analyzed GBs on CZTSSe absorbers with and without Lithium doping. The samples were measured in a N_2_ flow-box and had been soaked in DI water prior to the measurements. Li-free samples showed mostly downward band bending whereas the Li-doped ones exhibit predominantly upward band bending. Solar cell efficiencies fabricated from Li-doped samples are slightly higher than for Li-free samples (10% vs. 8% efficiency). Jiang *et al*.^9^ measured CZTSe GB properties in an argon filled glovebox without DI water soak. Preferential downward band bending at the GBs, similar to their high performance CIGSe absorbers (grown at NREL) have been measured. As a side note, we would like to emphasize that CIGSe GB properties are also under intense debate and no widely accepted model is currently available^4,5^. Sardashti *et al*.^10^ showed that the hydrazine processed CZTSSe absorbers exhibit mostly upward band bending (measurements were conducted in air after air-annealing followed by a NH_4_OH rinse). Erkan *et al*.^11^ showed that the GBs show mostly upward band bending after cleaning with KCN and NH_4_OH. Measurements where conducted in air shortly after the chemical cleaning step.

Quantitative Photoluminescence is a powerful tool to the investigate solar cell materials since it allows to extract directly the quasi Fermi-level splitting (QFLs) and thereby the junction voltage. It has been used by a number of people to study the optoelectronic properties of the absorber layers(see for example^12,13^). Recently it has been shown that it is also sensitive to changes in the surface properties. As shown by Lee *et al*. a thin Al_2_ O_3_ layer on top of the CZTSSe leads to an increase in PL yield and in V_*OC*_^14^. Consequently, the method can be used to study different surface passivation strategies. However, as we will see in the simulation part of this paper, the interface recombination velocity needs to be large in order to influence the QFLs, which is a bulk property.

The experimental results introduced in the preceding paragraphs are summarized in Table 1. Downward and upward band bending at the GBs are reported in literature and there is no general agreement which GB properties are more beneficial for solar cells. Possible reasons could be different measurement protocols (measurements in inert gas versus air; chemical cleaning vs as-grown/oxidized samples), process-dependent GB properties or varying GB compositions due to extrinsic doping or different sulfur to selenium ratios.

**Table 1:** Summary of the literature results on GB properties.

Reference	absorber type	GB work function [meV]	band bending
^7^	CZTS	−200	downward
	CZTSSe	−120	downward
^8^	CZTSSe no Li	≈−250	downward
	CZTSSe + Li	≈150	upward
^9^	CZTSe	≈−150	downward
^10^	CZTSSe	60−100	upward
^11^	CZTSe + KCN	15–20	upward

The GB values indicated here correspond to changes in the work function at the GB compared to the bulk interior. The approximate values given for some results are extracted from line-scans, whereas the other ones are taken from the authors text directly.

In this manuscript we will discuss in detail how NH_4_OH chemical cleaning influences the work functions of CZTSe surfaces and GBs. In CIGSe it is known that NH_4_OH is increasing the workfunction of the absorber since oxides and segregation of foreign atoms are removed due to this treatment^15^. We will investigate this treatment in CZTSe together with an in-depth analysis of the GBs as a function of surface treatment. We will not restrict the analysis to individual GBs but we will analyze a large number of different GB CPDs. This is important, as it has been shown that the GB symmetry changes preferential segregation of foreign atoms (Na, K, O)^16^ which may very well have an impact on the work functions of the GBs. We would like to emphasize that reports on the influence of alkalies in CZTSe are not restricted to segregation to the grain boundaries. Amongst others, it has been shown that Na can enhance the grain growth^17^, an improvement in the solar cell efficiency has been reported^18,19^ and changes in the sub bandgap states^20^ are observed.

We finally link the changes in surface properties to changes in the QFLs measured with calibrated Photoluminescence and to numerical simulations. We can show that our CZTSe absorber layers exhibit a large surface recombination velocity and that type inversion on as-grown or chemically treated surfaces is necessary for high quasi-Fermi level splitting.

## Results and Discussion

### Photoluminescence & KPFM results

**Figure 1:**
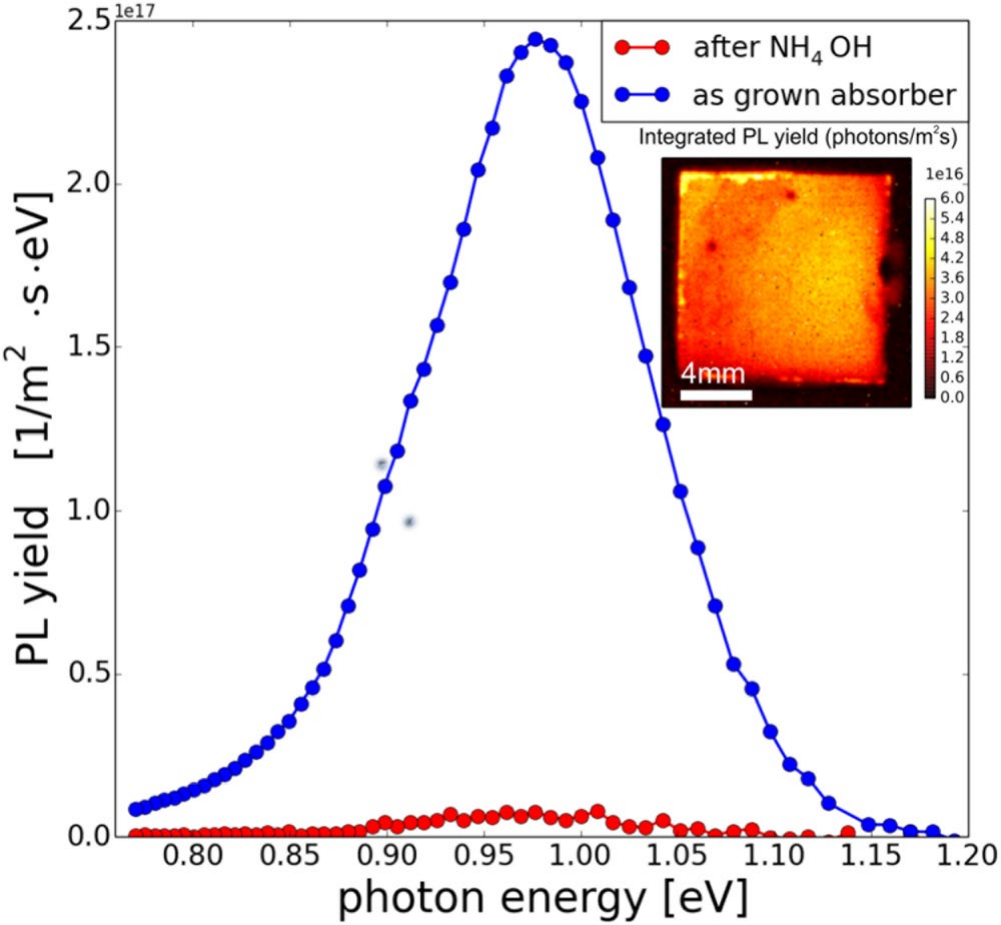
Photoluminescence yield of a CZTSe absorber after growth (blue) and after NH_4_OH etching (red). The inset shows the spectrally integrated photoluminescence map of the as-grown absorber under investigation.

In Fig. 1 photoluminescence imaging results are presented for a CZTSe absorber before and after NH_4_OH etching. The inset shows a spectrally integrated PL image of the absorber layer before the treatment. On the as-grown sample we observe a broad PL transition and we deduce a QFLs of 450 meV, which is a typical value for state of the art CZTSe absorbers. After NH_4_OH etching, which is a part of the CdS buffer layer deposition, we observe a strong quenching of the PL yield. This behavior is at first glance unexpected and undesirable. On the other hand, CIGSe absorber do not show this behavior and the PL yield stays almost identical after the NH_4_OH treatment (see supplementary information).

The modifications of the ammonia treatment are likely to be limited to the near surface region since it is well-known that the etching rate of CZTSe in NH_4_OH is negligible^11^.

**Figure 2:**
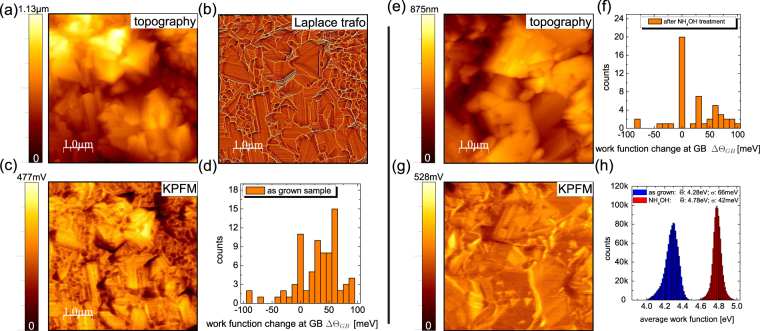
AFM and KPFM results of the as-grown (**a**–**d**) and NH_4_OH etched absorber (**e**–**h**). The topography is depicted in (**a**,**e**), the Laplace transformation of the topography shown in (**a**) is shown in (**b**), the contact potential difference map in (**c**,**g**), the work function changes at the GBs according to equation 1 in (**d**,**f**) and in (**h**) the average work functions of the as grown and NH_4_OH etched absorber.

In order to shed more light on the observation we carried out KPFM measurements. A typical result for an as-grown CZTSe absorber is shown in Fig. 2(a–d). The topography is depicted in Fig. 2(a), the Laplace transformation of the topography is depicted in Fig. 2(b), the contact potential difference map is shown in Fig. 2(c) and the grain boundary work function changes ${{\rm{\Delta }}{\rm{\Theta }}}_{GB}$ are shown in Fig. 2(d). The Laplace transformation of the topography is used to identify the exact positions of the GBs. It has been shown via correlative KPFM/Electron backscattering diffraction^5^ that this is a very effective method to identify GB positions. However, there are also some limitations of this method. The authors of ref.^5^ could show that twin GBs in CIGSe are difficult to be identified from the Laplace transformation, since they may not always show topographic features. Our results are therefore representative for high angle GBs and an unknown fraction of twin GBs. Having identified individual GBs, we can measure changes of the work function at GBs, denoted as ${{\rm{\Delta }}{\rm{\Theta }}}_{GB}$. This is accomplished by drawing line profiles perpendicular to the boundaries, as identified in the Laplace transformation of the topography and measuring the work functions left of the GB Θ_*l*_, at the GB Θ_*GB*_ and right of the GB Θ_*r*_. The changes in the GB work function can then be calculated with equation 1.

1 $${{\rm{\Delta }}{\rm{\Theta }}}_{GB}={{\rm{\Theta }}}_{GB}-(\frac{{{\rm{\Theta }}}_{l}-{{\rm{\Theta }}}_{r}}{2}+{{\rm{\Theta }}}_{r})$$

In our current setup we are not able to measure variations smaller than ±15 mV, which is typical for AM modulated KPFM in inert gas. This value has been deduced on an atomically flat HOPG surface (see supplementary information). Larger (lower) work functions at the GBs, compared to the grain interior, is indicative of negative (positive) charge carrier accumulation resulting in upward (downward) band bending. In the present case we observe a clear tendency for upward band bending. The median value ${\rm{\Delta }}{\mathop{{\rm{\Theta }}}\limits^{ \sim }}_{GB}$ of the measurement performed on the as-grown sample is 36 meV.

It is well known that Na segregates at the surface and at the grain boundaries in CZTSe^16^. We assume that this is also the case in the absorbers investigated here. Consequently the NH_4_OH treatment will change the near surface region by removing alkalies and we anticipate strong changes in the work function and in the surface region of the GBs.

The analysis of the same sample after NH_4_OH is presented in Fig. 2(e–g). The surface looks cleaner and many of the “small grains” have disappeared. This observation is not limited to the image presented here but rather general. We observe a clear facet depending CPD contrast and the number of GBs that exhibit upward band-bending is reduced. The median value ${\rm{\Delta }}{\mathop{{\rm{\Theta }}}\limits^{ \sim }}_{GB}$ reduces from 36 meV to 8 meV.

The average work function $\bar{{\rm{\Theta }}}$ (defined as the work function value averaged over multiple KPFM images and many different grains) increases compared to the as-grown case, as depicted in Fig. 2(h). The chemically cleaned surfaces exhibit a higher work function and the work function variations also reduce as illustrated by the standard deviation of the distribution. The histograms in Fig. 2(h) have been computed from the images presented in Fig. 2(c,g).

This observation is not surprising since we have to assume that a Na covered surface exhibits a lower work function (The work function of Na is 2.75 eV according to ref.^21^). Our results are also in good agreement with investigations of CIGSe absorbers, where as-grown samples exhibit a reduced work function, compared to *in-situ* prepared or chemically cleaned surfaces^22^.

From the present analysis we can conclude that GB work function variations decrease upon NH_4_OH etching and that the surface is cleaned, which results in a higher average work function. At this point we already see that different cleaning/measurement protocols can strongly influence the KPFM results. We do see a substantial change of the GB work functions ${\rm{\Delta }}{\mathop{{\rm{\Theta }}}\limits^{ \sim }}_{GB}$ as a result of an NH_4_OH cleaning step. This result is certainly one reason for the discrepancies between the different reports available in literature (cf. Table 1).

However, from the information presented so far we do not know which of the measured changes induce the reduction of the QFLs as depicted in Fig. 1. We therefore decided to heat the absorber layer in air to promote oxidation and, at sufficiently high temperature, a segregation of Na from the CZTSe bulk to the surface. In the following, we show the results for a set of heating/cleaning cycles on one CZTSe absorber layer.

**Figure 3:**
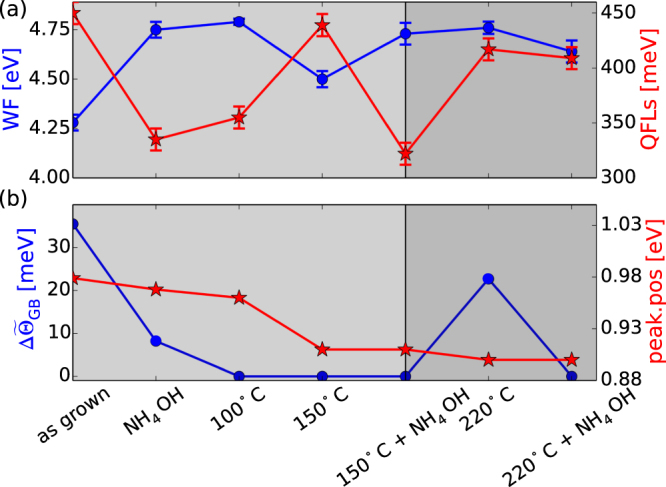
(**a**) Work functions (WF), Quasi Fermi-level splitting (QFLs), (**b**) Photoluminesecence peak position and median value of the grain boundary work function changes ${\rm{\Delta }}{\mathop{{\rm{\Theta }}}\limits^{ \sim }}_{GB}$ of the same absorber after different surface treatments as labeled on the x-axis.

A summary of all the treatments, the evolution of the average work function $\bar{{\rm{\Theta }}}$ and of the QFLs together with the median value of the GB work function changes ${\rm{\Delta }}{\mathop{{\rm{\Theta }}}\limits^{ \sim }}_{GB}$ and the changes in PL peak position are depicted in Fig. 3. The KPFM measurements for 100 °C, 150 °C, 150 °C + etching, 220 °C and 220 °C + etching are summarized in the supplementary information. The first two data points have already been presented and discussed in Fig. 2. A 100 °C air annealing step does not change the sample significantly and $\bar{{\rm{\Theta }}}$ stays constant and the QFLs stays as low as for the NH_4_OH treated surface. At that stage we do not see any preferential upward band bending anymore (${\rm{\Delta }}{\mathop{{\rm{\Theta }}}\limits^{ \sim }}_{GB}$ = 0). Most of the analyzed GBs do not have band bending or the work function changes are below the resolution limit of the KPFM system.

At 150 °C annealing, the work function decreases accompanied by a sharp increase of the QFLs. The value is close to the value of the as-grown absorber. In addition to the changes in work function and QFLs we observe that the PL peak position decreases, which is a direct consequence of the order/disorder phase transition in CZTSe^23,24^. This result shows that the as-grown absorbers have a larger degree of ordering than the air-annealed samples, which can be traced back to a faster cooling rate in the later case. We performed a low temperature air annealing on these type of samples too and observed that the PL peak shifts to higher energies as ordering increases in complete agreement with other reports (see supplementary information).

The grain boundary potentials ${\rm{\Delta }}{\mathop{{\rm{\Theta }}}\limits^{ \sim }}_{GB}$ are still very low and we do not see substantial band bending. A 5 minutes etching step in NH_4_OH recovers the “etched state”, i.e. a high work function and low PL yield. At 220 °C we do see a recovery of the PL yield but this time the work function stays high in contrast to the 150 °C case. We do see an increase in the grain boundary potentials (${\rm{\Delta }}{\mathop{{\rm{\Theta }}}\limits^{ \sim }}_{GB}$ > 0) and preferential upward band bending. However, the variations are rather small. It is more intuitive to compare the histograms of all the GB potentials which can be found in the supplementary information. A subsequent NH_4_OH etching removes the potentials at the GBs but this time the QFLs and the average WF do not change significantly anymore.

From the observations we can define two different regimes which are color coded in Fig. 3 (light gray and dark gray). In the first regime we do see that etching increases the WF but reduces QFLs. In the second regime (above 220 °C) we do have a relatively high WF with a high QFLs, which cannot be changed by etching anymore. We do see that the PL peak position is lowest in region 2, which corroborates that we actually reached the highest possible amount of disorder (for our cooling rate).

In the following, we will try to give a consistent explanation for the observations. We will first discuss the results in literature and see if any of the available models explain our observations.

Sardashti *et al*.^10^ proposed that air annealing leads to preferential oxidation of the GBs via the formation of SnO_*x*_. SnO_2_ is a high bandgap material (*E*_*g*_ ≈ 3.5 eV^25^) which likely forms a large type I conduction band offset with CZTSe. The type of band bending due to SnO_*x*_ cannot be inferred from the bandgap since the work function depends strongly on the exact oxygen content *x* of the SnO_*x*_ compound^26^. In our case we do see that air annealing (for the highest temperature) induces some upward band bending, in accordance with Sardashti *et al*. However, the most important changes due to heating are linked to variations of the average work function, which arises from a change of the surfaces of the individual grains. Such large variations in the average work function of a polycrystalline film cannot be explained solely by changes in the grain boundaries, since the GB area is too small compared to the total surface area. It is therefore unlikely that a change in the GB properties alone can explain our results.

Another explanation has been given by Kim *et al*.^27^. They showed via high resolution electron microscopy techniques that substantial amounts of oxygen can be incorporated into the CZTSe matrix via a replacement of Se. This leads to an increase in bandgap (from 0.98 eV (Cu_2_ZnSnSe_4_) to 1.48 eV in the case of Cu_2_ZnSn(Se_3_O)). The hole barrier increases but the conduction band offset to the GBs is type 2, i.e. a cliff. They also propose that a very thin oxygen rich kesterite is forming on top of the absorbers which might act as an additional hole barrier. This explanation could be compatible with our results since we do see a permanent change of the average work function after 220 °C air annealing.

On the other hand, Larsen *et al*.^28^ reports that, upon air annealing, the near surface region shows an increase in the Zn content. SnO_*x*_, ZnO, and Na_2_S(e)O_3_, Na_2_S(e)O_4_ are measured right after the air annealing, which can be removed by an NH_4_OH etching step. These measurements are also in line with our results. Air annealing leads to oxide phases which may lower the average work function. NH_4_OH etching removes these oxide phases and a higher work function (the one from the CZTSe) is recovered. At high temperatures the material properties in the near surface region changes irreversibly. In the case of Larsen *et al*. the most prominent change was an increase in the Zn content as measured by XPS after etching the surface.

The reports by Kim *et al*., and Larsen *et al*. are in partial agreement with results by Haight *et al*.^29^ who showed that, after air annealing the surface is enriched in oxygen, depleted in Cu and enriched in Zn. Interestingly, they showed that the oxide phases, after the high temperature air annealing step can be removed by a NH_4_OH treatment. From this result we conclude that the observations presented in Fig. 3 cannot originate from an oxygen-rich surface, since the QFLs is insensitive to NH_4_OH in the second region of Fig. 3. Haight *et al*. also showed that the PL yield is drastically increased after air annealing, in complete analogy to our results.

In summary, the reports in literature suggest that, for our measurements the formation of a more Cu-poor and Zn-rich surface is most likely responsible for the observed changes in work function and QFLs. An air annealing temperature of at least 200 °C needs to be used in order to permanently modify the near surface region and this modification is stable against an NH_4_OH solution.

### Numerical simulations

**Figure 4:**
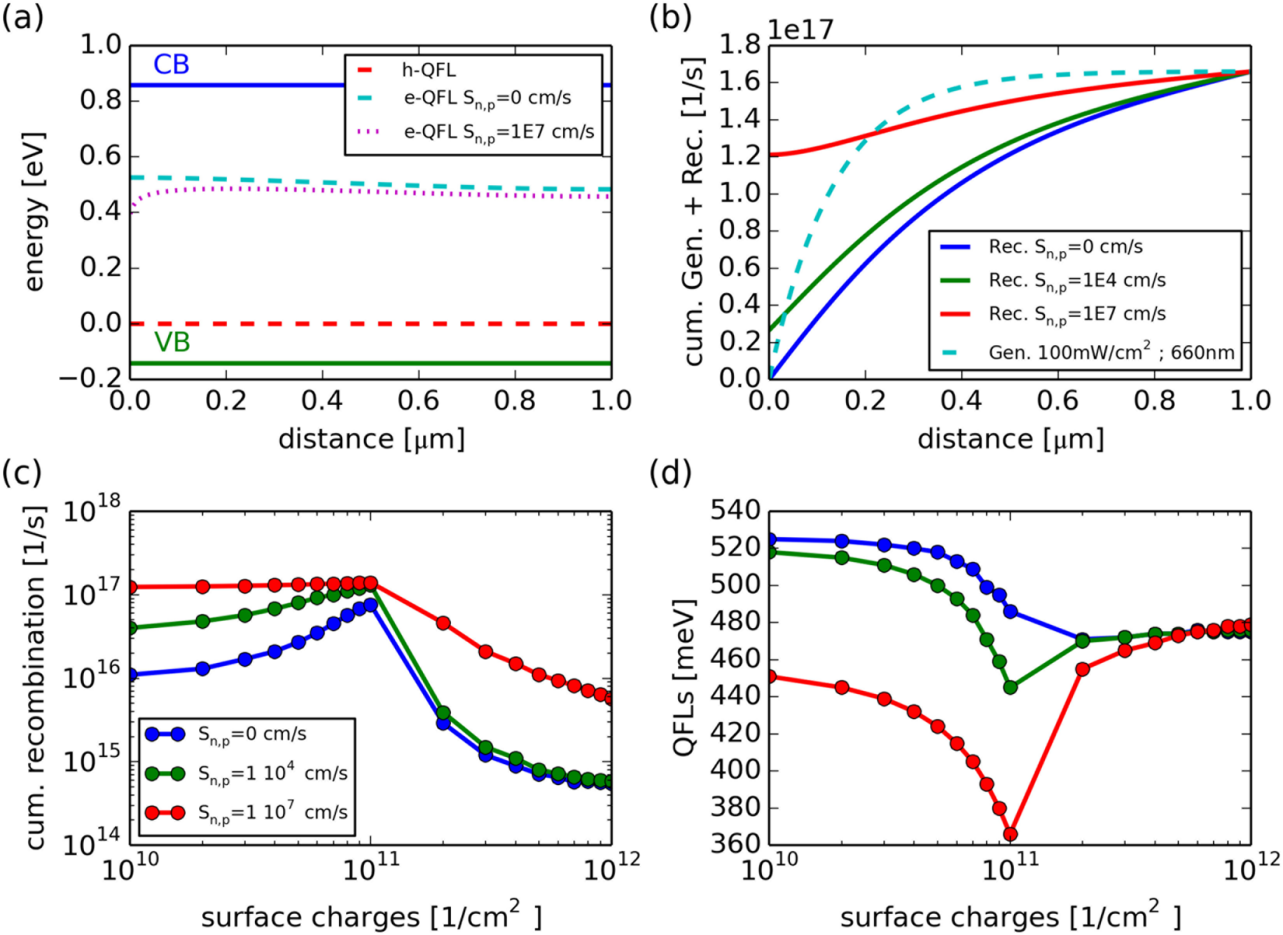
PC1d-simulations illustrating the effect of a high surface recombination velocity *S*_*n,p*_ and positive surface charges. (**a**) Band diagram of the CZTSe absorber under monochromatic illumination. The valence band (VB), the conduction band (CB) and the electron and hole quasi Fermi-levels (e(h)-QFL) are shown for two different *S*_*n,p*_. (**b**) Cumulative generation and recombination for three different *S*_*n,p*_. (**c**) Cumulative recombination measured from the surface to a 30 nm depth as a function of surface charges for three different *S*_*n,p*_. (**d**) QFLs deduced as the minimum distance between the electron and hole QFLs as a function of positive surface charges.

The question remains, how to link the work function changes to the changes of the QFLs in region I of of Fig. 3. Here, we have seen that air annealing reduces the WF but increases the QFLs. An NH_4_OH etching step recovers the original state of high WF and low QFLs. Based on the results by Larsen *et al*. and Haight *et al*. we assume that this effect is triggered by soluble oxides. In order to understand how a decrease in WF can explain an increase in QFLs we carried out numerical simulations with PC1D^30^ and the results are presented in Fig. 4.

We use the following set of parameters to model the absorber. The CZTSe absorber exhibits a thickness of 1 *μ* m, a bandgap of 1 eV, a dielectric constant of 10 and a refractive index of 3.6. The level of p-type doping is set to 1 10^16^ cm^−3^, electron mobilities are set to 50 cm^2^ V^−1^ s^−1^, and hole mobilities to 20 cm^2^ V^−1^ s^−1^ ^31^ and we use an experimentally determined absorption coefficient^32^. The bulk carrier lifetimes (holes and electrons) have been set to 1 ns^33^, the surface recombination velocity is varied from 0 cm/s to 1·10^7^ cm/s and positive surface charges are varied from 1·10^10^ to 1·10^12^ cm^−2^ during the simulations. In the simulation we always change the surface charges for one specific surface recombination velocity.

The absorber is illuminated with 100 mW/cm^2^, 660 nm monochromatic light to be close to the experimental conditions (The difference in illumination intensities between experiment and simulations (190 mW/cm^2^ compared to 100 mW/cm^2^) will only increase the simulated QFLs by 16 meV). Throughout the simulations we only change surface properties (surface recombination velocity and surface charges) and all bulk properties stay constant.

In Fig. 4(a) the band diagram of the absorber is shown for two cases. In the case of a low surface recombination velocity the electron and hole QFLs are rather flat. The small change towards the back can be attributed to the Lambert-Beer absorption profile used in the simulation. In the case of high surface recombination velocities the e-QFLs is reduced in the near surface region. Recombination is strongly affected by this effect, as shown in Fig. 4(b) where the cumulative recombination and generation rates are depicted. The indicated values show total integrated quantities (number of recombined(generated)charge carriers per second) from the absorber surface (at x = 0 *μ*m) up to a certain depth given on the x-axis. A strong increase at the front of the absorber is indicative of a high surface recombination. The integrated generation profile is shown as a dotted line and the cumulative recombination rates are shown for three different surface recombination velocities. It is clear from the graph that a high recombination velocity leads to a strong recombination current in the near surface region since the cumulative recombination is high at small distances from the surface.

Surface charges lead to a modification of the band structure in the near surface region. The work function is changed via a change in the Fermi-level position. In the following we want to analyze the effect of the band bending, due to surface charges, on recombination. The integrated (cumulative) recombination is a direct measure of how many carriers recombine from the surface up to a certain distance in the bulk. In the following, we define the distance to be 30 nm away from the surface. All the trends discussed in the following are independent of the chosen distance. Only the exact numbers vary to a certain extent, which is not important since our description is not quantitative.

As illustrated in Fig. 4(c), the recombination current increases strongly up to a charge of approximately 1·10^11^ 1/cm^2^ (in the case of a high ${S}_{n,p}$). Increasing the number of charges further, leads to a strong reduction of the recombination rate. The reason for this drastic change is that the surface becomes inverted (i.e. electrons are majorities at the surface of a p-type semiconductor). This inversion pushes the recombination into the bulk of the absorber and the surface becomes less important. In Fig. 4(d) the QFLs, defined as the minimum separation of the electron and hole QFL is shown as a function of the surface charges. In the case of a high surface recombination velocity the QFLs reduces until inversion is reached followed by a sharp increase of the QFLs by almost 150 meV. This observation is not correct for the situations where the surface recombination velocity is small. In that case the QFLs is higher for the non-inverted case since surface charges lead to an increase of the minority carrier (electron) population in the near surface region, which increases recombination.

The presented simulations offer a possible explanation for the observations presented in Fig. 3. We have to assume a defective CZTSe surface with a large surface recombination velocity (${S}_{n,p}$ $> 1\cdot {10}^{5}$). We need such a high *S*_*n,p*_ to account for the large dependence of the QFLs on surface charges. We assume that surface charges, as used in the simulations are responsible for the experimentally observed changes in the WF. The assumption of a high surface recombination velocity in CZTSe is in line with other reports, as for example ref.^14^. Air annealing leads to the formation of oxides such as SnO_*x*_, ZnO, and Na^2^ S(e)O_3_, Na_2_S(e)O_4_ which reduce the work function and which leads to an inversion of the surface. The PL yield is high and the work function is low. After surface cleaning with NH_4_OH, the inversion is lifted and the QFLs is strongly reduced. Air annealing above 200 °C leads a permanent change of the surface due to the formation of a more Zn-rich and Cu-poor surface, as described in literature. This permanent change of the surface is probably reducing the recombination velocity which is beneficial for solar cells. The explanation given here does not include changes in GBs as we did not see any systematic changes of the QFLs variations with GB CPDs. The beneficial effect of air annealing will vanish as soon as the surface recombination velocity is substantially reduced. This is clearly visible in Fig. 4(d). In order to then further improve the device performance the minority carrier lifetime has to be increased (a parameter that has not been varied in this set of simulations). As a final note we would like to be emphasize that the high surface recombination velocity is not reduced by the CdS buffer layer. We have systematically investigated this on many different absorbers processed by our sequential sputtering followed by selenization process and we could never observe an increase of the PL yield after CdS buffer layer deposition.

## Conclusions

We have shown via a combination of Kelvin Probe Force Microscopy and Photoluminescence imaging that the absorber quality, defined by a high QFLs, changes if the the work function of the absorber is modified. This finding has been corroborated by numerical simulations. In order to reproduce the experimental observations we have to assume a quite large surface recombination velocity (in the range of 10^5^–10^7^ cm/s). In that case, surface charges have a strong influence on QFLs since they influence the recombination in the surface region. Such high values of *S*_*n,p*_ are not good enough to compete with high performance thin film devices. As an example, it has recently been shown that surface recombination velocities as low as 10 cm/s can be achieved in organic/inorganic hybrid perovskites^34^. Moreover, we could show that annealing above 200 °C leads to a permanent modification of the work function which is probably connected to a change in the surface composition (either oxygen and/or a change in the metal ratios). This modification makes the surface insensitive to NH_4_OH treatments. We do not see a direct correlation of the grain boundary potentials with the QFLs variations. Most of the upward band bending, that we have measured on as-grown samples is removed during the first NH_4_OH cleaning step. Based on our findings we propose that the current bottleneck in CZTSe is the high surface recombination velocity. This also explains why many reports in literature have shown improvements via air annealing and/or modification of the buffer layer.

## Methods

### Absorber synthesis

The Cu_2_ZnSnSe_4_ films have been prepared by a sequential process. Metallic stacked precursors are grown via DC sputtering from high purity Zn/Cu/Sn targets on Mo coated soda lime glass (800 nm). The elemental ratios of the precursors are: Cu/Sn = 1.8, Zn/Sn = 1.1 and Cu/(Zn + Sn) = 0.86. In the next step, the samples are selenized in a tube furnace with 60 mg Se pellets in a graphite box. We have used a two step process where first the samples are annealing under vacuum at 350 °C for 10 minutes. In the second step, the background pressure is increased to 100 mbar N_2_ and the temperature is ramped to 490 °C for an additional 20 minutes. The samples are cooled down naturally. Solar cells fabricated from this precursor batch yield device efficiencies of 6–7%. Glow Discharge Optical Emission Spectroscopy (GDOES) measurements presented in the supplementary information corroborate that Na is present in our films and there is some preferential segregation to the surface.

### Kelvin Probe Force Microscopy

Kelvin Probe Force Microscopy (Anfatec Instruments AG) has been measured in a resonance enhanced single pass mode. We use amplitude modulation (AM) KPFM in an inert gas environment (<1% humidity, <1% O_2_). AC voltages of 0.5 V- 1 V have been used throughout the study and the bias has been applied to the sample while the tip was grounded. PtSi cantilevers with a resonance frequency of 75 kHz (force constant 2.8 N/m) and a tip radius of 25 nm have been used. The work function (WF) of all tips have been calibrated on highly oriented pyrolytic graphite^35^. We are aware of the fact that we will not measure the true work function, as obtained for a clean surface prepared under ultra high vacuum conditions^22^. The work functions measured here are therefore considered to be effective work functions, where the true WF is reduced by a constant value, which describes the influence of the inert gas exposure. Moreover, due to the long range nature of the electrostatic force, the influence of the cantilever geometry needs to be taken into account^36^. A discussion of this effect on our rough samples together with some reference measurements are presented in the supplementary information. Image analysis has been carried out with WSxM^37^. All the analyses have been carried out on raw data without image processing. The grain boundaries have been chosen randomly (by hand) based on the Laplace transformation of the topography image. Regions with artifacts, as discussed in the supplementary information where not considered. In the topography measurements presented in the Fig. 2a “sobel-type” edge contrast matrix filter has been used to improve the visibility.

### Photoluminescence (PL) Imaging

PL imaging was carried out using a custom setup described elsewhere^38,39^. In the present case we used a 660 nm Laser to illuminate the complete sample with approximately 190 mW/cm^2^. The QFLs has been deduced from the evaluation of the high energy slope of the PL spectrum^38,40^. The QFLs given in the manuscript are spatially averaged values (sample size ≈1 × 1 cm^2^).

### Surface treatments

The NH_4_OH treatment has been done in the following way. A fresh sample has been mounted on the AFM sample holder and electrical contact between the holder and the sample surface has been made with conductive silver lacquer. The drying process has been carried out in a glove-box. After the KPFM measurements on the untreated CZTSe surface, the sample has been removed from the AFM sample holder stage and a few drops of a 25 wt% NH_4_OH solution were put onto the sample surface. Due to surface tension the solution was confined on the surface and did not come in contact with the sample holder/glue. After 5 minutes at room temperature the sample was immersed into DI water and left in there for the transport to the AFM. The sample was then dried under a N_2_ stream and mounted into the AFM system, which was immediately purged with N_2_ to keep the air exposure as low as possible (always less than 3 minutes of air exposure could be achieved). Sample heating has been accomplished with a small ceramic heater under ambient conditions. A dummy absorber was placed on the hotplate where a Pt100 thermocouple has been connected to measure the temperature. Annealing to 100 °C has been accomplished via a fast ramping (≈60 s) to 100 °C followed by a 5 minute dwell time. For the 150 °C and 220 °C no dwell time was used, since the heating up to such high temperatures took considerably longer than for the 100 °C case. Consequently, the samples where only heated up to the desired temperature, which took approximately 5 minutes, followed by a cool down. The cooling down was done naturally. The temperature that we refer to in the present manuscript are sample temperatures measured with the Pt100 temperature sensor and not hotplate settings. The cooling rate during selenization was estimated to be 12 K/min (between 200–100 °C) whereas in air it was 53 K/min, i.e. a factor of 4.4 faster. This explains why there is substantially more Cu/Zn disorder in the films during the post selenization heat treatments compared to the as grown films.

## Electronic supplementary material

Supplementary information
