# Supplementary material for: High surface recombination velocity limits Quasi-Fermi level splitting in kesterite absorbers
Source: Sci Rep. 2018 Jan 30;8:1874. doi: 10.1038/s41598-018-19798-w (PMC5789844; doi:10.1038/s41598-018-19798-w)
Supplement: Supplementary file 1 — Supplementary information [file 41598_2018_19798_MOESM1_ESM.pdf]

Supplementary information: High surface recombination velocity limits Quasi-Fermi level splitting in kesterite absorbers

Alex Redinger*,†,‡ and Thomas Unold†

†*Department Structure and Dynamics of Energy Materials, Helmholtz-Zentrum Berlin für Materialien und Energie*

‡*Scanning Probe Micrsocopy Laboratory, Physics and Materials Science Research Unit, University of Luxembourg*

E-mail: alex.redinger@uni.lu

1 Analysis of the different surface treatments

The following figures show the surface morphology and the evaluation of the grain boundary work function changes for the different surface treatments that have been discussed in Figure 3 of the main manuscript.

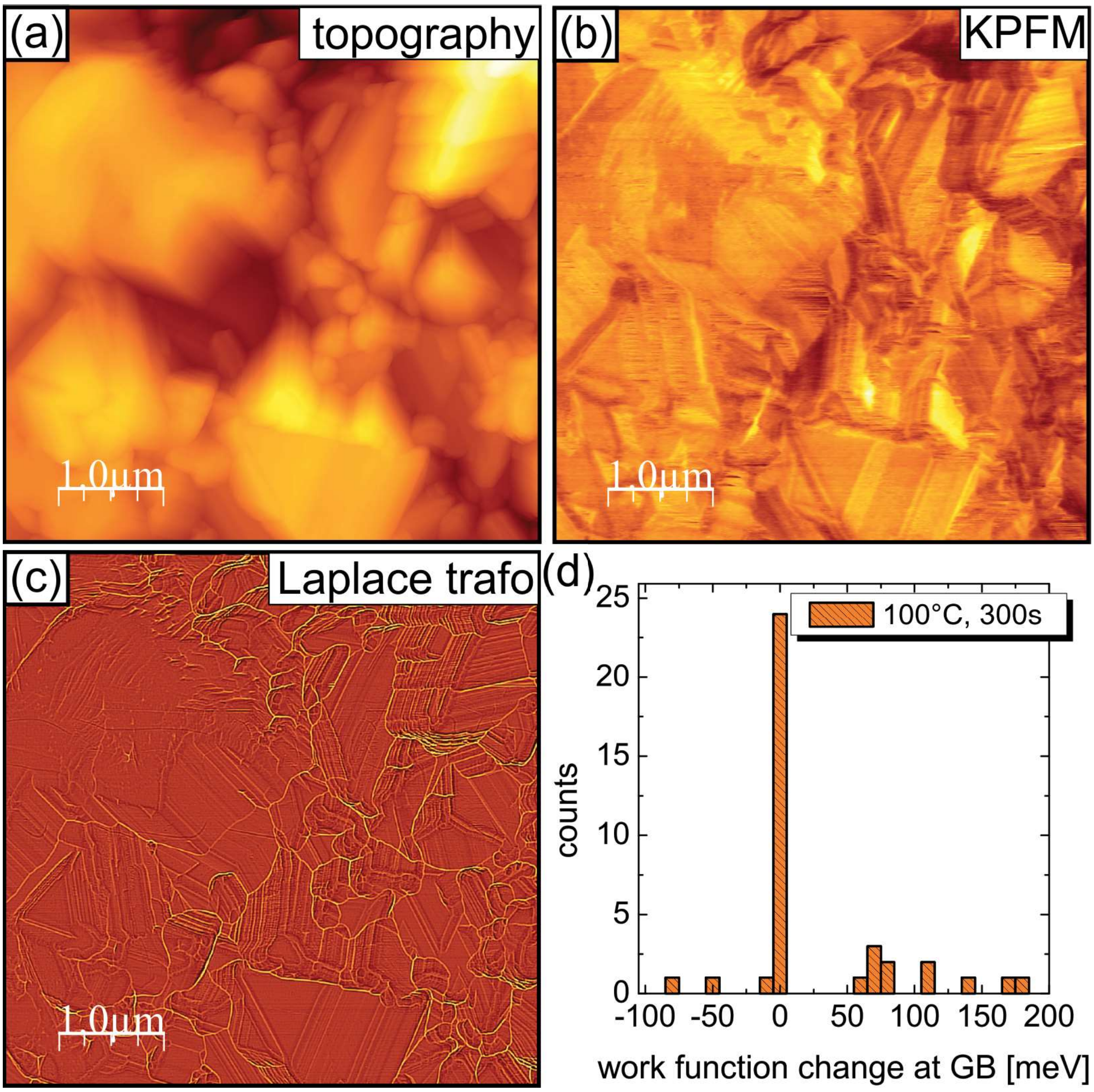

Suppl. Fig. 1: Air annealing 100 °C, 5 minutes; (a) Topography, (b) KPFM image, (c) Laplace transformation, (d) work function changes at GBs

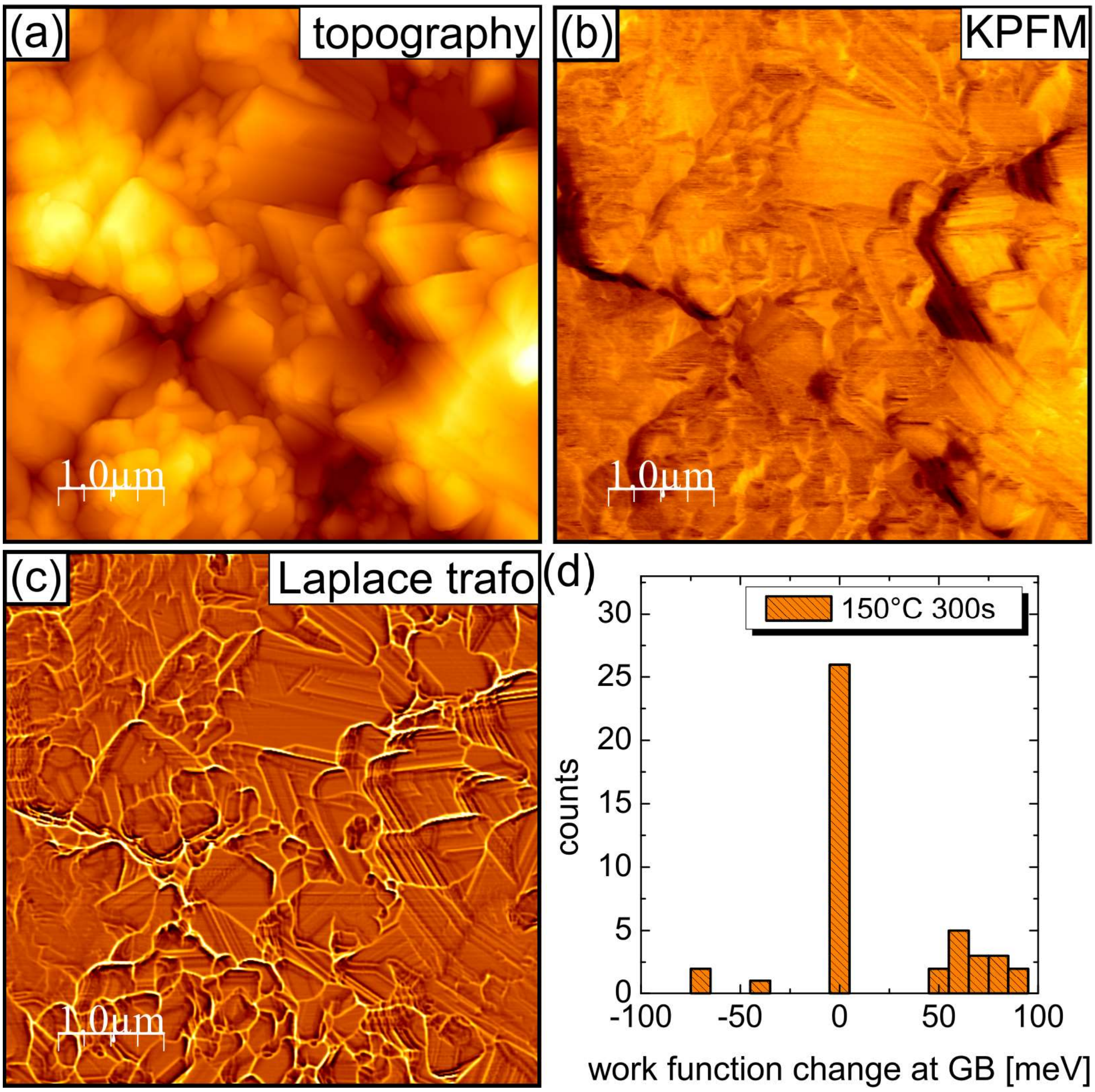

Suppl. Fig. 2: Air annealing 150 °C, 5 minutes; (a) Topography, (b) KPFM image, (c) Laplace transformation, (d) work function changes at GBs

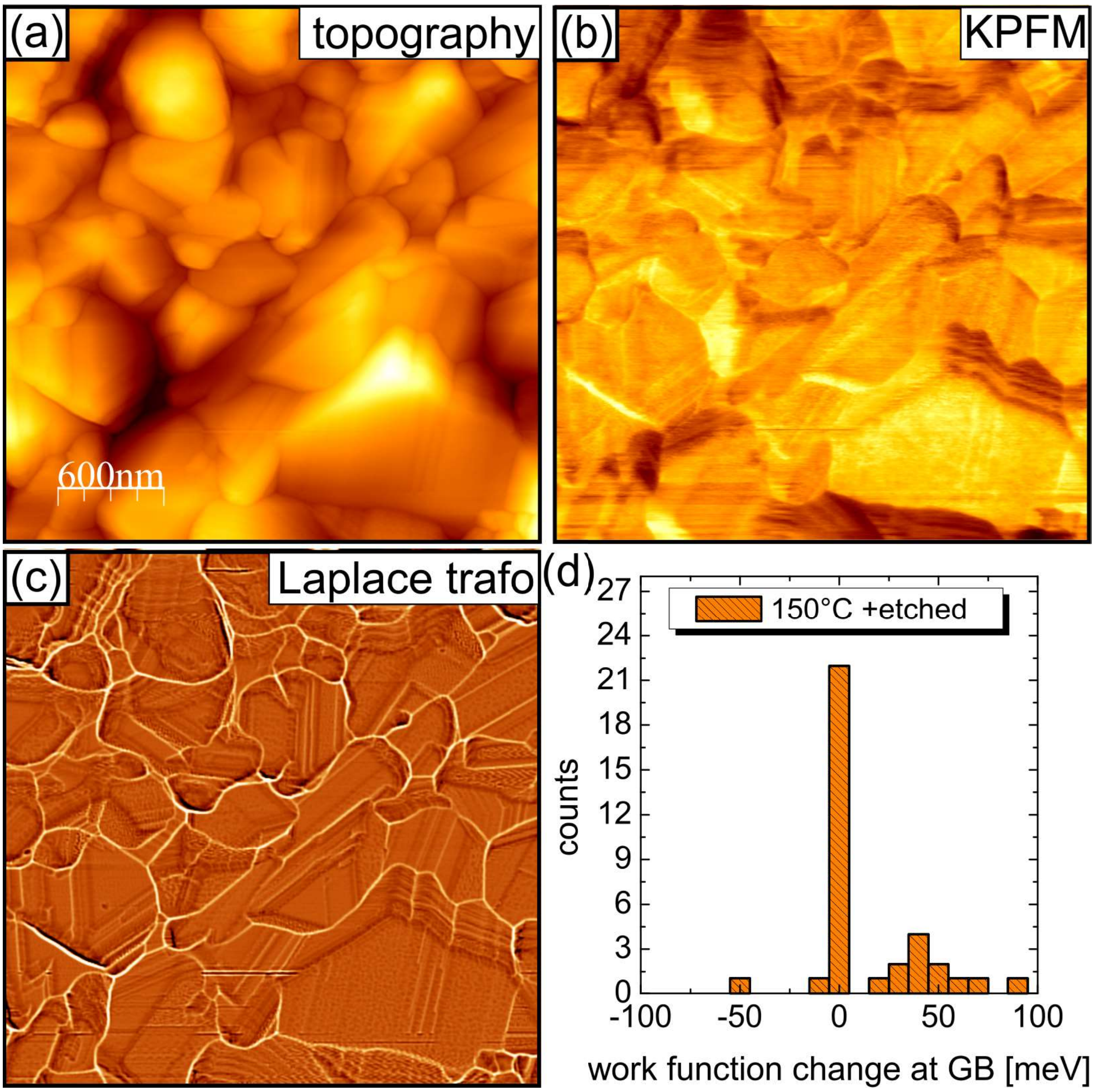

Suppl. Fig. 3: Air annealing 150 °C+NH_4OH; (a) Topography, (b) KPFM image, (c) Laplace transformation, (d) work function changes at GBs

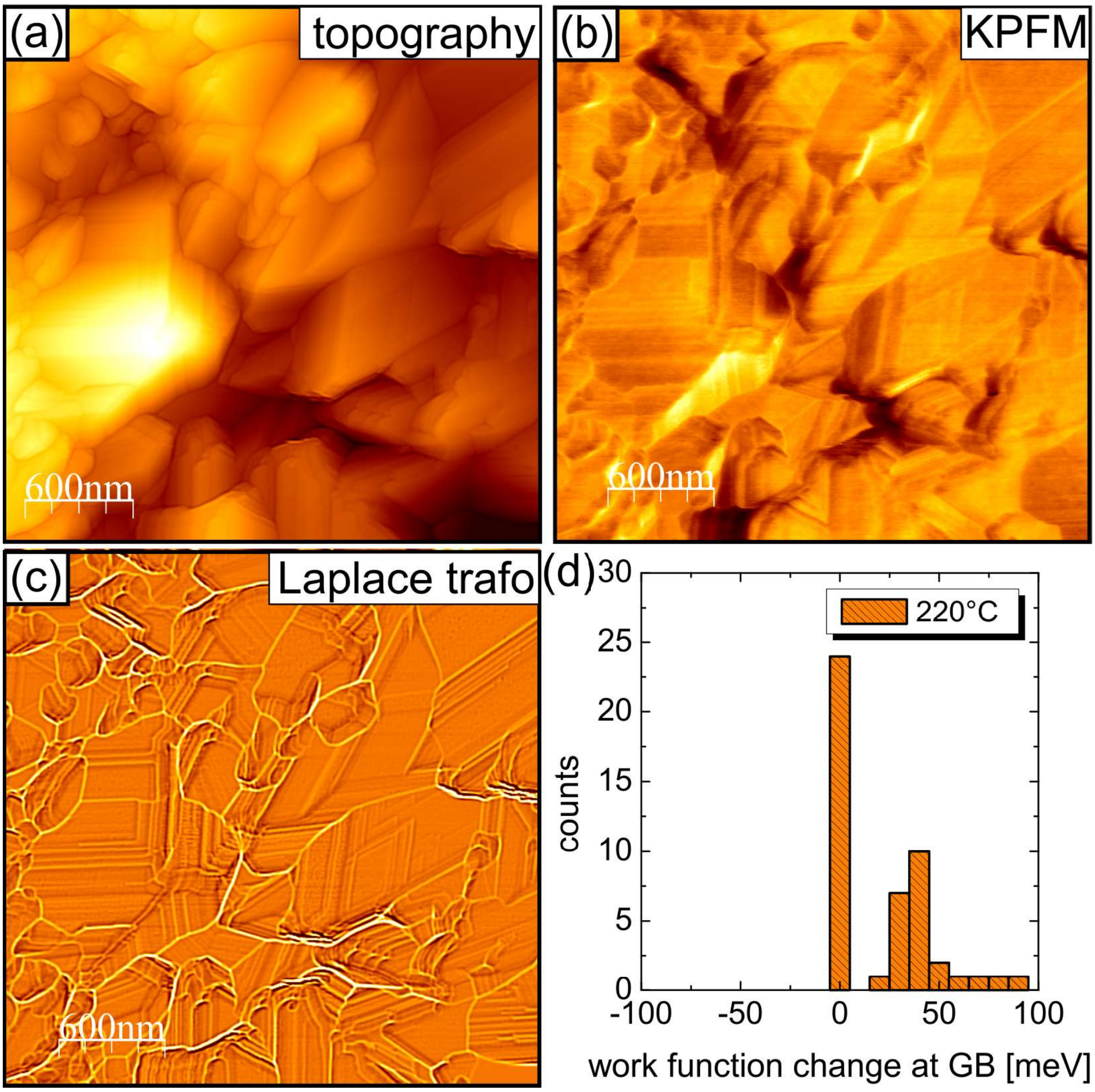

Suppl. Fig. 4: Air annealing 220 °C; (a) Topography, (b) KPFM image, (c) Laplace transformation, (d) work function changes at GBs

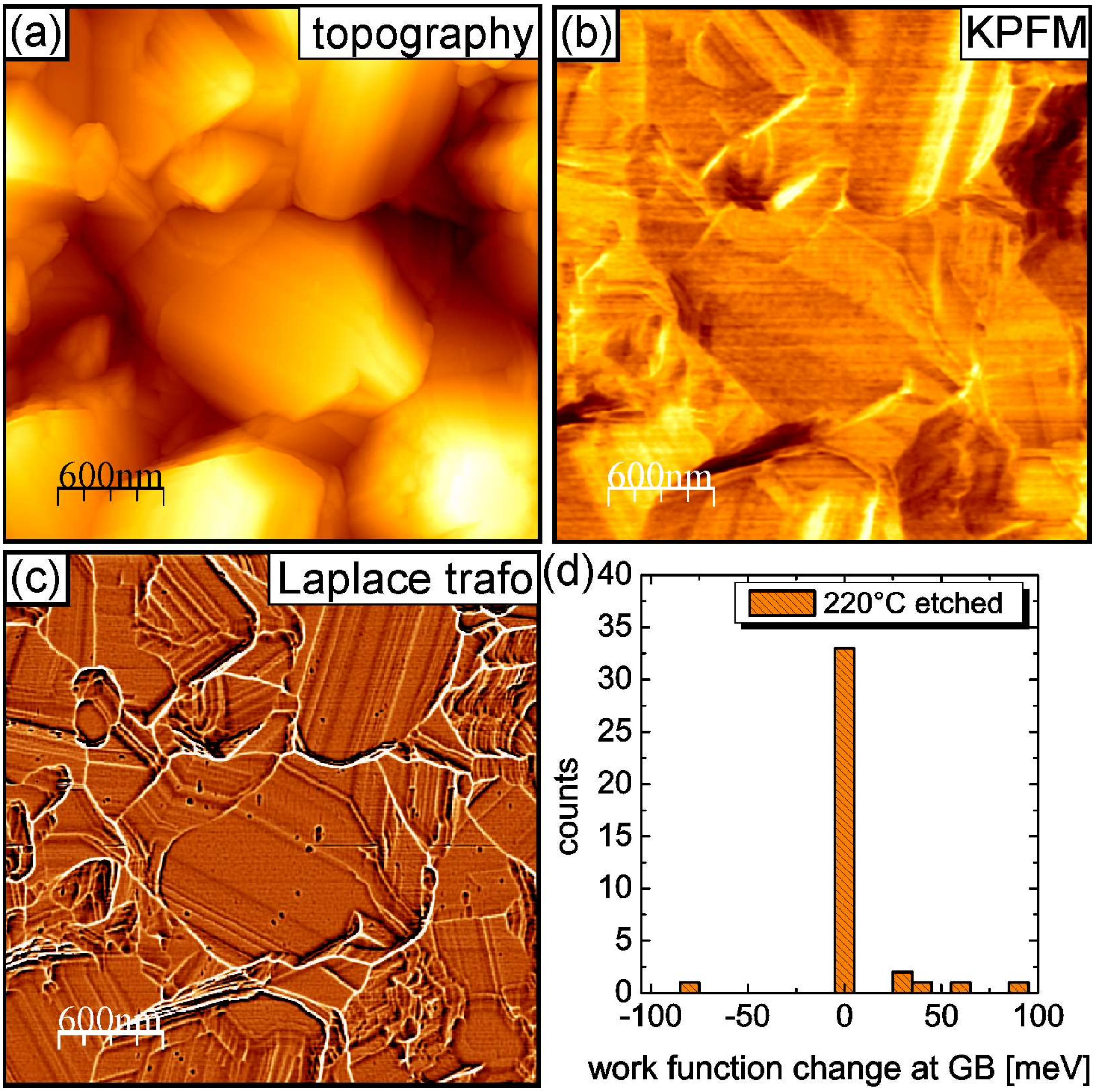

Suppl. Fig. 5: Air annealing 220 °C+NH_4OH; (a) Topography, (b) KPFM image, (c) Laplace transformation, (d) work function changes at GBs

2 Comparison of the NH_4OH surface cleaning on CIGSe

In Figure 6 the changes in PL yield before and after NH_4OH etching on a CIGSe sample is presented. The changes are extremely small in contrast to the kesterite case (compare Figure 1 in main text). The double peak observed in Figure 6 is not due to two transitions but due to interference fringes. The surface roughness on the coevaporated CIGSe absorbers is much smaller than on the kesterite absorbers..

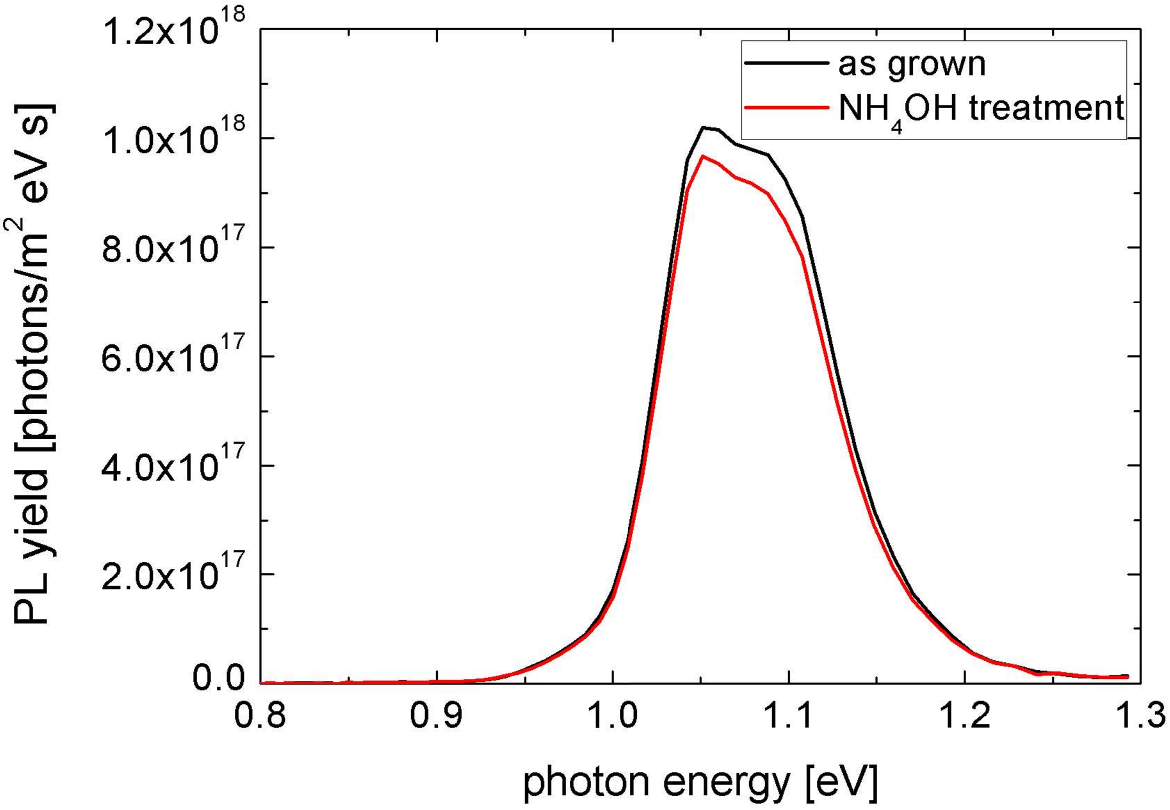

Suppl. Fig. 6: PL measurements on a a 15 % CIGSe absorber layer before and after NH4OH etching. The treatment has been carried out under exactly the same conditions as for the kesterite case

3 Na distribution within the CZTSe absorber

Figure 7 depicts Glow Discharge Optical Emission Spectroscopy (GDOES) measurements of the Na, C and Mo distribution within the CZTSe film. The carbon concentration is increased towards the front layer which points to surface contamination (as a result of the air exposure). The increase of the Mo signal after ≈ 200 s indicates the CZTSe/$MoSe_2$ interface. The smearing out of this interface is attributed to surface roughness. The Na distribution increases towards the front surface. The decrease within the Carbon rich layer is neglected. The accumulation of the Na at the kesterite/$MoSe_2$ layer is also commonly observed. The measurements presented here underline that the assumption in the main manuscript (accumulation of Na to the near surface region) is well justified.

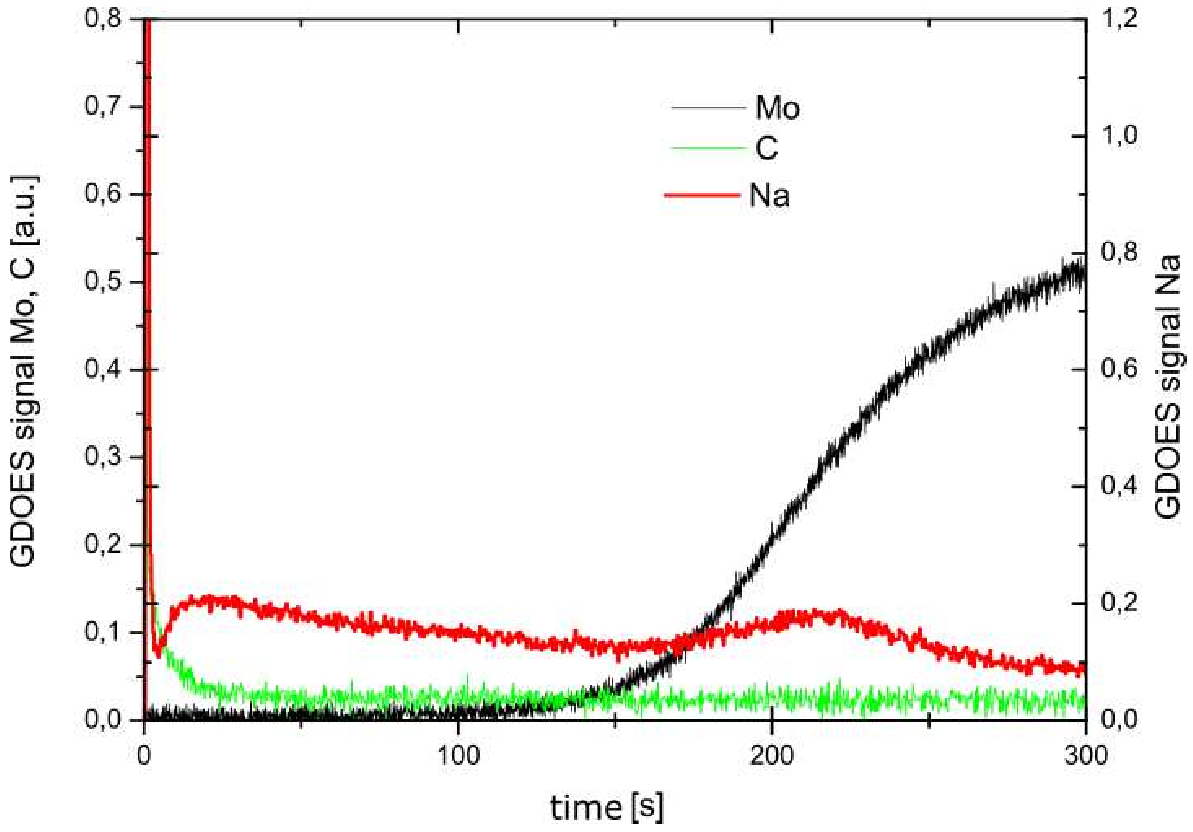

Suppl. Fig. 7: GDOES measurements performed on a similarly produced kesterite absorber. The Mo, C and Na signal is presented as a function of sputter time which is proportional to the sputter depth.

4 Topological artefacts in the KPFM signal

KPFM is prone to topological artifacts due to the long range nature of the electrostatic force. Consequently the shape of the complete cantilever influences the measurements. Most of the signal originates from the tip apex. Especially on rough samples the influence of the cantilever cannot be neglected. The artifact is shown in Figure 8. The red circles indicate positions which are exhibit a rather larger change in surface topography. In the KPFM signal these positions are shown as a dark shadow. The feature is not considered to be real and these regions have not been evaluated.

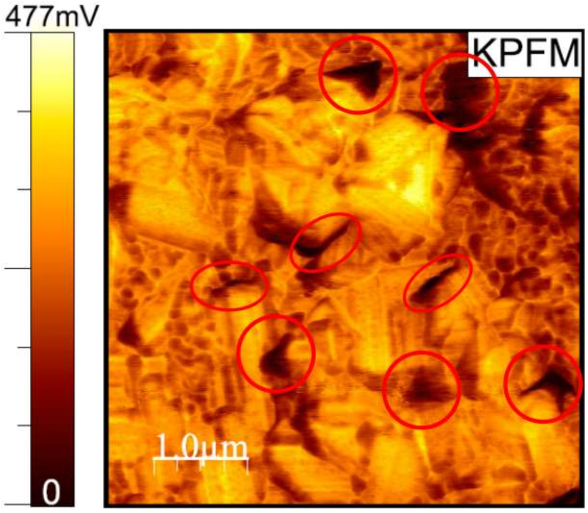

Suppl. Fig. 8: KPFM measurement of a CZTSe absorber layer. The red circles highlight regions which are influenced by the cantilever geometry and which have to be discarded in the evaluation of the grain boundary potentials.

However, it has to be emphasized that the measurements are indeed meaningful for height variations where the influence of the cantilever stays constant, i.e. small changes in height. This is shown in Figure 9. The sample consists of patches of Au grown on Pt. The topography is depicted in (a) and the CPD in (b). It is clearly visible that we have a very clear response with a lower work function of Au compared to Pt as expected from literature. The workfunction literature values scatter substantially since different crystallographic orientations have different work functions.[1] Values for Au are in the range of 5.3 to 5.5eV whereas

the value for polycrystalline Pt is approximately 5.6 eV. In the present case we measure approximately 100 meV CPD different. This value is probably to low due to the influence of the cantilever as discussed for example in the following references.[2,3] However, it is clear that the the variations in the workfunction are correctly measured with our present setup despite the fact that the absolute value is wrong.

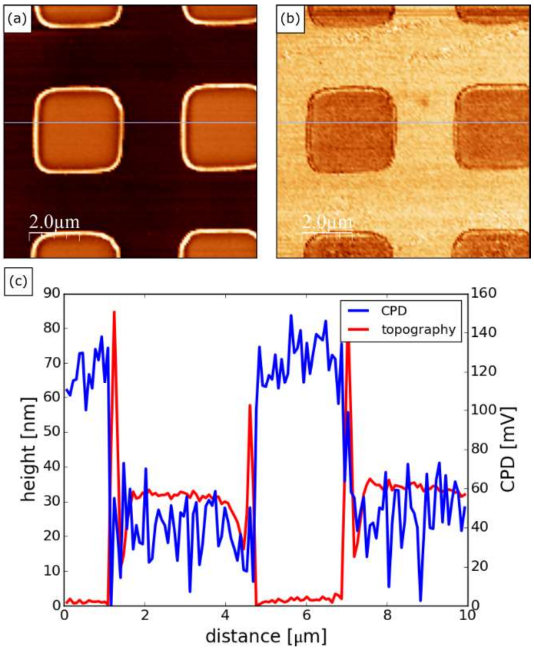

Suppl. Fig. 9: Au on Pt reference sample.(a) topography, (b) CPD measurement

This has important consequences for the grain boundary potentials deduced in the present study. The values that are measured are too small and they need to be taken as a lower limit for the real grain boundary potentials. However, the trend (upward or downward band bending) is reflected correctly in our measurement. More details can be found in the following reference.[4]

5 Resolution limit of the KPFM setup

In order to get a lower bound for the energy resolution limit of our KPFM setup we measured the CPD on a HOPG samples that was cleaved freshly. The results are presented in Figure 10

where the topography and the CPD values are presented together with a horizontal linescan. We deduce a RMS nose of 18meV and peak to peak variation of approximately 35 mV. From this measurements we deduce that we are unable to measured variations in the CPD value which are smaller than +/- 15meV.

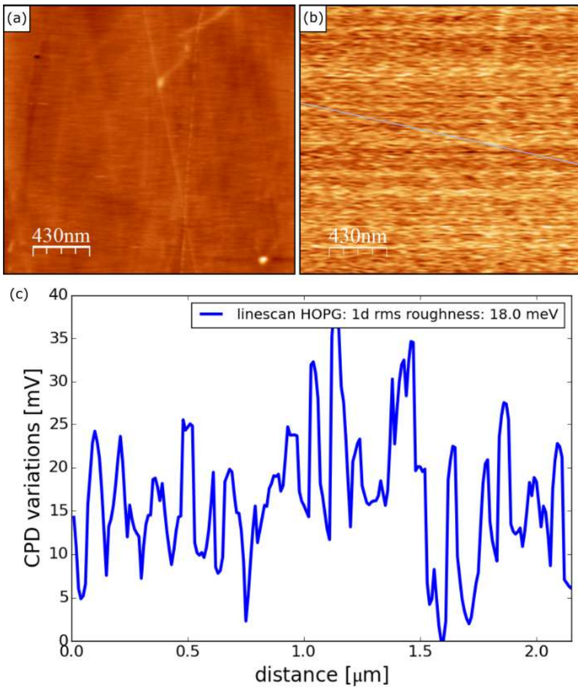

Suppl. Fig. 10: HOPG sample (a) topography, (b) CPD measurement, (c) representative linescan

6 Order/disorder

After the heating and cleaning treatments described in the manuscript the sample has been used to test how a low temperature heat treatment changes the Cu/Zn disorder. This is presented in figure 11. In complete agreement with literature, the PL peak position increases as ordering increases. An interesting observation is that the PL yield stays almost constant, which shows that recombination is not affected by the heat treatment. The bandgap increases, which also increases the QFLs and the final V_{OC} of the devices. However, the V_{OC} deficit stays constant.

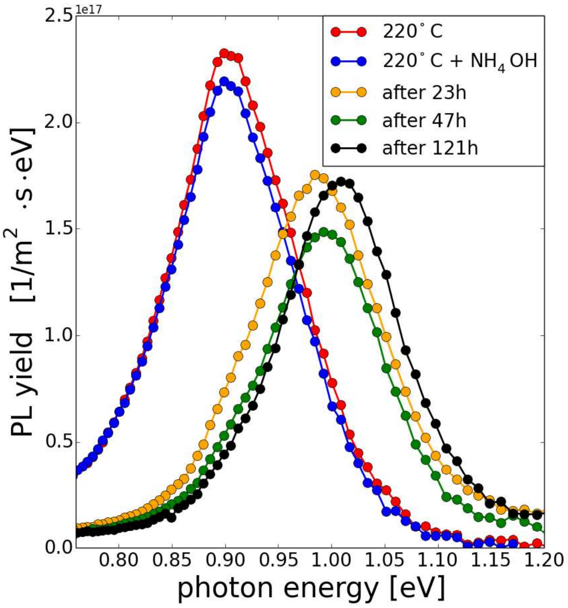

Suppl. Fig. 11: CZTSe sample heated to 50 °C (hotplate temperature). At the given times the samples has been cooled down to room temperature and measured in the imaging setup to determine the PL peak position and the PL yield.

References

(1) Haynes, W. M. *CRC handbook of chemistry and physics*; Taylor & Francis.

(2) Glatzel, T.; Sadewasser, S.; Lux-Steiner, M. C. Amplitude or frequency modulation-detection in Kelvin probe force microscopy. *Applied Surface Science* **2003**, *210*, 84–89.

(3) Zerweck, U.; Loppacher, C.; Otto, T.; Grafström, S.; Eng, L. M. Accuracy and resolution limits of Kelvin probe force microscopy. *Physical Review B - Condensed Matter and Materials Physics* **2005**, *71*, 1–9.

(4) Baier, R.; Leendertz, C.; Abou-Ras, D.; Lux-Steiner, M. C.; Sadewasser, S. Properties of electronic potential barriers at grain boundaries in Cu(In,Ga)Se_2 thin films. *Solar Energy Materials and Solar Cells* **2014**, *130*, 124–131.
